# Design and optimization of sheller for ginkgo nut: A study about multifunctional ginkgo nut sheller

**DOI:** 10.1371/journal.pone.0276139

**Published:** 2022-10-27

**Authors:** Minji Liu, Jiannan Wang, Ni Wang, Huanxiong Xie, Huijuan Zhang, Haiyang Shen, Xuemei Gao

**Affiliations:** 1 Nanjing Institute of Agricultural Mechanization, Ministry of Agriculture and Rural Affairs, Nanjing, Jiangsu, China; 2 College of Engineering, Nanjing Agricultural University, Nanjing, Jiangsu, China; 3 Jiangsu Academy of Agricultural Sciences, Nanjing, Jiangsu, China; 4 Hunan Agricultural Equipment Research Institute, Changsha, Hunan, China; Tongji University, CHINA

## Abstract

At present, ginkgo nut shellers have many problems, such as low shelling rate and high damage rate. To address these problems, a multifunctional ginkgo nut sheller was designed. The equipment had functions for shell breaking, shelling, and separation of shell from the kernel. The influencing factors of the shelling process were analyzed. A three-factor two-level response surface test was conducted, and mathematical models for the response surface were established. The influence of each factor on the operation quality was analyzed, and a combination of parameters was optimized. The experimental results showed that the factors in the order of importance of their influence on shelling rate was as follows: feeding rate, chainplate speed, and shelling gap. The factors in the order of importance of their influence on damage rate was as follows: shelling gap, chainplate speed, and feeding rate. The results of the interaction analysis conducted showed that the interaction between the chainplate speed and shelling gap had the highest significant effect on the shelling rate, followed by the interaction between the feeding rate and chainplate speed. The interaction between the chainplate speed and shelling gap had a significant effect on damage rate. The interaction between the other factors had no significant effect on shelling and damage rates. The optimal combination of parameters was as follows: the feeding rate was 30.2 g/s, the chainplate speed was 0.48 m/s, and the shelling gap was 12.3 mm. The optimal combination was employed, and the validation test resulted in a shelling rate of 98.02% and damage rate of 4.45%. The relative error between the measured and theoretical values was less than 5%, indicating that the models were reliable. This study can provide a reference for subsequent research on shelling equipment of ginkgo nut.

## Introduction

Ginkgo is widely planted in China, with a planting area of approximately 400,000 ha. Ginkgo nuts are rich in crude fats, nuclear proteins, minerals, vitamin C, riboflavin, and a variety of amino acids. They are highly nutritious and have high edible and medicinal values [1, 2]. The average annual production of ginkgo nuts is approximately 500,000 tons, accounting for more than 90% of the total global production [3]. The output value is approximately 30 billion yuan [4], and the industrial scale is still expanding. Shelling is the premise for the utilization of ginkgo nuts. The conventional manual shelling is labor intensive and inefficient, which cannot meet the industrial demand. Therefore, the demand for mechanized shelling is increasing [5–9].

In China, the existing research on nut shelling mainly focuses on peanut, while the research on ginkgo nut shelling is much less. In the early days, scholars carried out some relevant research. Yuan et al. (2002, 2004) conducted experiments on ginkgo nuts after drying and freezing using a roller-plate sheller. They determined the main factors and optimal combination of factors that affected the operation quality of the sheller [10, 11]. Zhu et al. (2006, 2008) designed a roller-plate ginkgo nut sheller. Using the improved roller-plate structure, the graded and infrared dried ginkgo nuts were tested. The test shows that the improved sheller increased the operation performance [12, 13]. Liu et al. (2004) designed and tested a roller-plate ginkgo nut sheller, and the test results showed that the equipment can replace manual shelling and improve production efficiency [14]. However, the shellers are all roller-plate type. They only had a shell-breaking process, which was not sufficient for complete separation of shell and kernel. There were problems with low shelling rate and high damage rate. Moreover, Zhang et al. (2012) obtained the best process parameters for a drum-grid ginkgo nut sheller by investigating the influence of the rotor speed, drum diameter, and grid-gap on the shelling quality. The equipment broke the ginkgo nut shells through the interaction between the drum and screen strip [15]. However, due to the lack of shelling process, it had a low operation quality which could not meet the requirements for practical application.

Research on nut shelling in other countries is mostly focused on cashew nut, peanut, and other varieties. Research on ginkgo nut shelling is relatively few, and there is no special shelling equipment for ginkgo nut. Suda et al. (2006) developed a ginkgo nut machine by transforming a small rice mill. During operation, ginkgo nuts were broken by two drums with different rotational speeds. Cracked ginkgo nuts hit the disc under the drum, separating the shell from the kernel. Then, a cleaning fan cleaned and discharged the separated shell. The equipment could replace manual operation and substantially improve work efficiency [16]. Harless (1953) and Van (1996) invented a universal nut shelling machine, which can perform shelling operations for various types of nuts, including ginkgo nut. It effectively reduced the operation intensity and improved the shelling efficiency [17, 18]. However, the general equipment cannot meet the requirements of high-quality shelling of ginkgo nut, and problems, such as poor adaptability, poor shelling effect, and work quality, need to be addressed.

Thus, the existing studies on nut shelling mainly focus on peanut and cashew nut, while the studies on ginkgo nut shelling are much less. The ginkgo nut is irregularly fusiform. The shell is hard and the kernel is full and easy to break. After breaking, the kernel often adheres to the shell and is difficult to separate [5, 11, 13]. The characteristics of ginkgo nut are quite different from peanut and cashew nut. The characteristics of the material to be shelled determine the structure and process flow of the sheller, so it is difficult to learn from the existing research results. The existing ginkgo nut sheller has few structural forms, and most of them are roller-plate type [10–14]. There are the problems such as low shelling rate and high breakage rate, which can not meet the actual operation requirements [10]. It is necessary to improve the operation quality [14]. At present, researches on novel shelling structure and corresponding technological process are seldom seen. Therefore, it is necessary and challenging to innovate and design a high-performance sheller. In addition, there are many factors that affect the performance of sheller. In order to optimize the operation performance, it is necessary to optimize these factors. Response surface methodology (RSM) is an effective test method for optimizing process conditions. It is widely used in experimental studies of other types of shellers [19, 20]. RSM is also often used when it is costly and time-consuming to collect experimental data from expensive simulation models [21, 22]. We assume that the application of Box-Behnken design (BBD) method will help us simplify the experiment and improve the efficiency. RSM and multi-objective optimization will be used to obtain better data, which will help us determine the optimization results quickly and improve the experimental accuracy. The expected results of this study would provide a practical sheller for ginkgo producers and insights on improving operation performance.

## Structure and working principle of sheller

**Fig 1:**
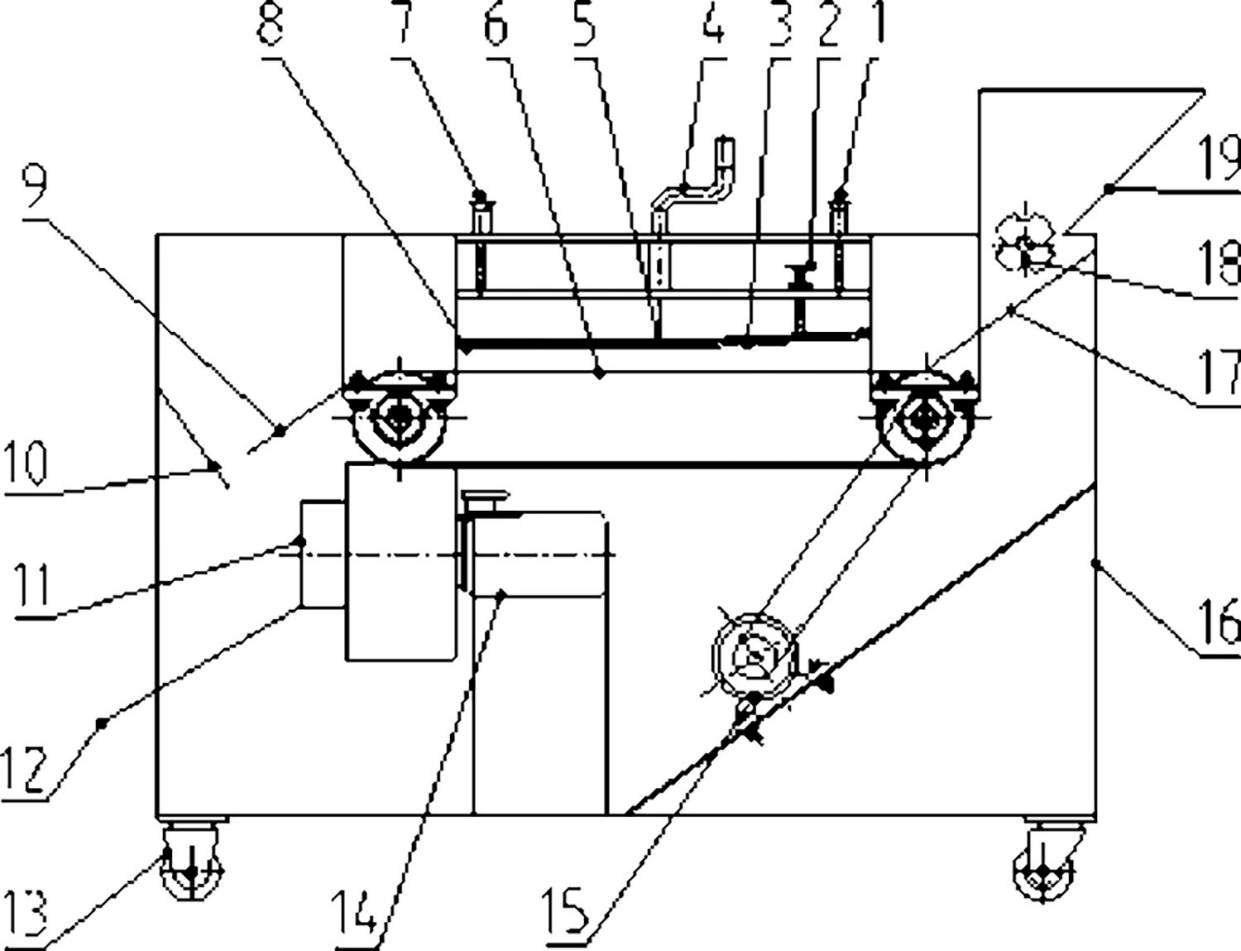
Schematic of the chainplate ginkgo nut sheller. 1. Positioning shaft Ⅰ. 2. Adjusting handle Ⅰ. 3. Hard rolling plate. 4. Adjusting handle Ⅱ. 5. Hinge. 6. Chainplate. 7. Positioning shaft Ⅱ. 8. Flexible rolling plate. 9. Discharge guide plate Ⅰ. 10. Discharge guide plate Ⅱ. 11. Impurity removal fan. 12. Discharge guide plate Ⅲ. 13. Ground wheel. 14. Motor Ⅰ. 15. Motor Ⅱ. 16. Frame. 17. Feeding guide. 18. Feeding roller. 19. Hopper.

**Fig 2:**
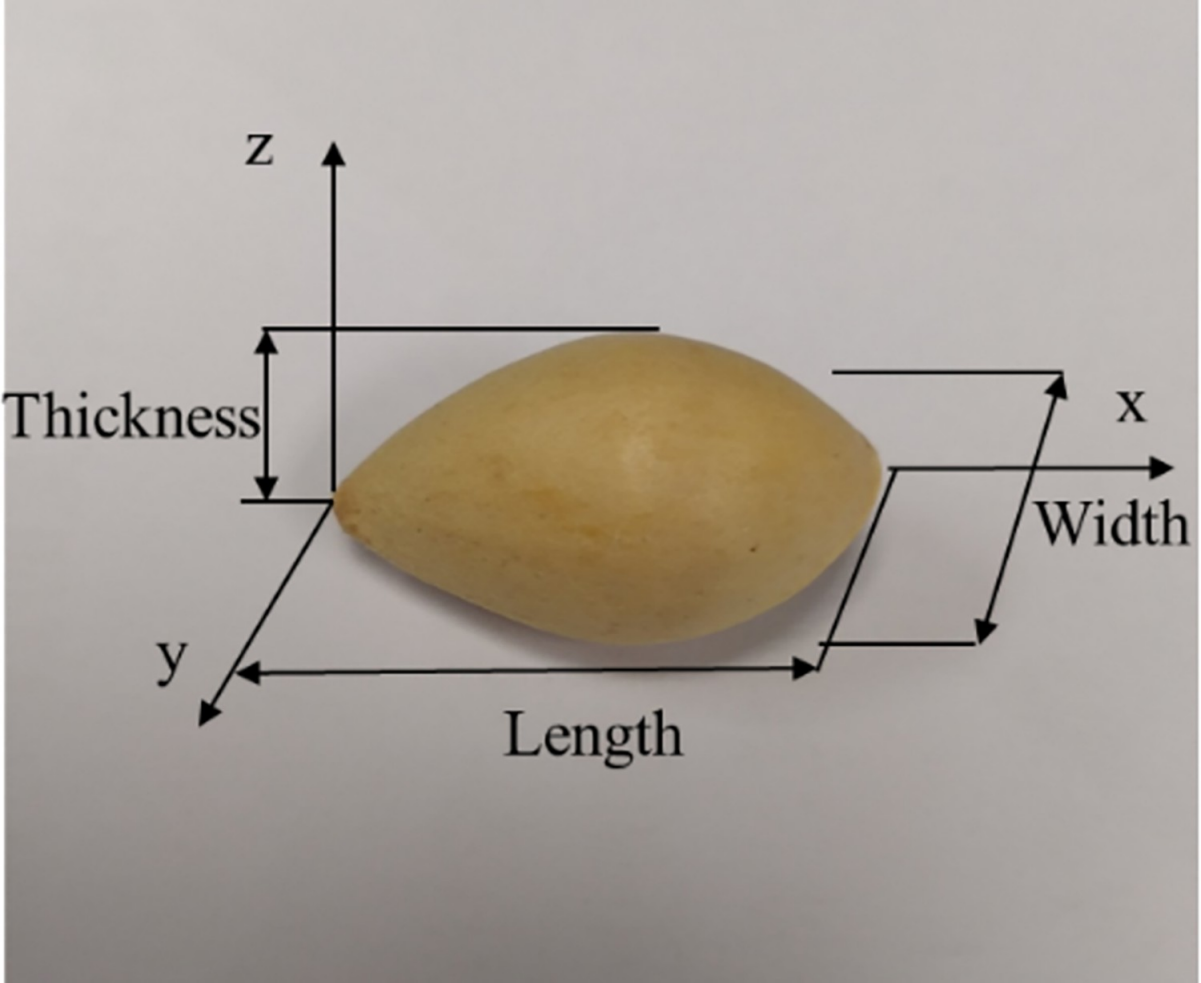
Ginkgo nut triaxial size.

The schematic of the chainplate ginkgo nut sheller is shown in Fig 1. It was mainly composed of a frame and feeding, shelling, impurity removal, discharging, and transmission devices. Multiple processes, such as shell breaking, shelling, and cleaning can be completed in one operation. During operation, the ginkgo nuts to be shelled are placed in the hopper and fed gradually by the feeding roller. They are guided to the metal chainplate by the feeding guide plate to ensure that the ginkgo nuts are evenly spread on the chainplate in a single layer in the thickness direction. The ginkgo nut triaxial size is shown in Fig 2.

The motor drives the metal chainplate to transport the ginkgo nuts forward. When passing through the hard rolling plate, the ginkgo shell is squeezed and broken. The cracked outer shell is gradually broken and separated from the inner kernel due to friction and rubbing action of the flexible rolling plate. The ginkgo nuts are squeezed and rubbed by the chainplate, hard rolled plate, and flexible rolled plate; thus, the shelling is more efficient. The shelled material is removed by the impurity removal fan to remove the broken shell, and the shell is separated from the kernel. The ginkgo nuts are then discharged from the discharge guide plate, and the shelling operation is completed.

## Design of key components

### Design of feeding device

The feeding device was composed of a hopper, feeding roller, and feeding guide plate. It is an important part of the ginkgo nut sheller. The function of the feeding device is to ensure reliable and smooth feeding of ginkgo nuts during operation. During the shelling operation, the ginkgo nuts to be shelled are placed in the hopper, and the nuts are fed continuously and smoothly through the rotation of the feeding roller. The feeding roller operates with the feeding guide plate to spread the ginkgo nuts evenly on the chainplate in a single layer in the thickness direction and improve the operational efficiency and quality of the equipment. To ensure smooth feeding, the inclination angles of the hopper slope and feeding guide plate should be greater than the sliding friction angle of the ginkgo nut. A previous test show that the sliding friction angle between the ginkgo nut and steel plate was approximately 30°. Therefore, the inclination angle between the inclined plane of the hopper and the horizontal plane was 45°. Due to the size of the equipment structure, the inclination angle of the feed guide plate was 35°.

**Fig 3:**
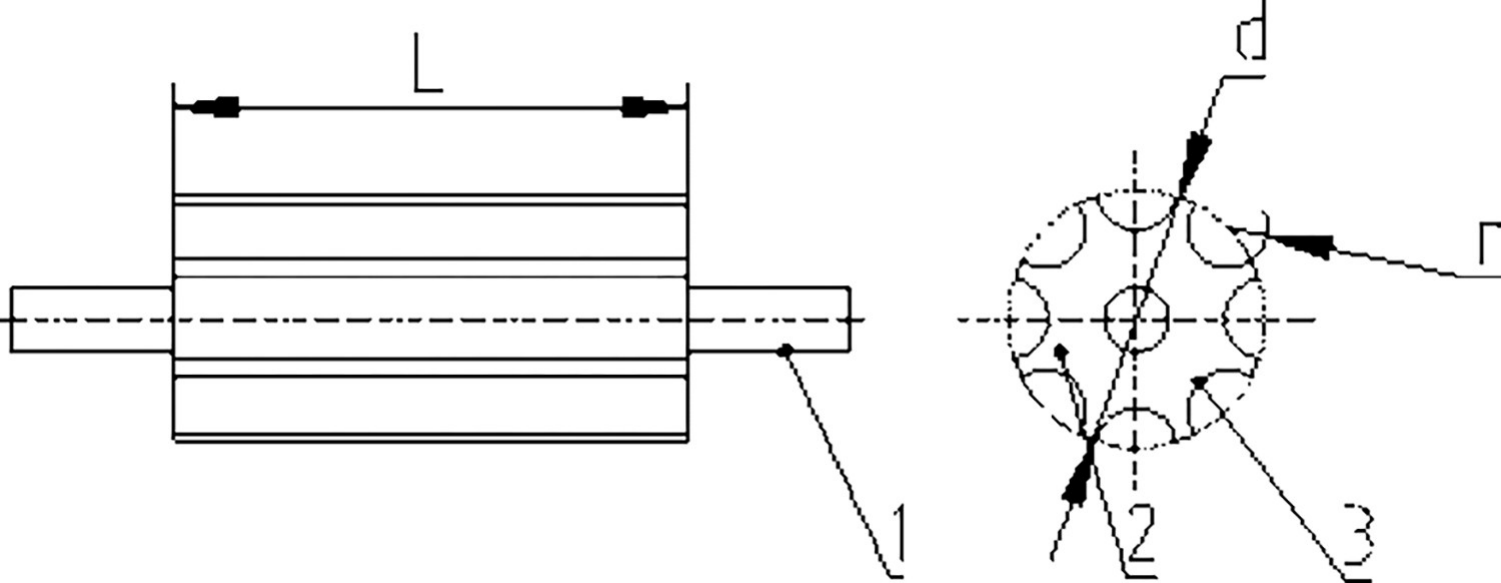
Schematic of the feeding roller. 1. Shaft. 2. Outer roller. 3. Groove.

The feeding roller is the key working part of the feeding device. It is an external groove wheel structure, as shown in Fig 3. It is driven and controlled by an independent motor and a speed regulating mechanism, and the speed can be adjusted in real-time according to the needs of the operation to ensure shelling quality.

The feeding amount of feeding roller *q* can be expressed as [23]

$$q=\pi dL\gamma (\frac{\alpha_{0}f_{q}}{t}+\lambda )$$ (1)

where *q* is the feeding amount per revolution, g/r;

*d* is the diameter of the feeding roller, cm;

*L* is the effective working length of the feeding roller, cm;

*γ* is the density of ginkgo nut, g/cm^3^;

*α*_*0*_ is the filling factor of the material in the groove;

*f*_*q*_ is the cross-sectional area of a single groove, cm^2^;

*t* is the groove pitch, cm; and

*λ* is the characteristic coefficient of the driving layer.

Based on the "Design Manual of Agricultural Machinery" [23] and the preliminary test, the diameter *d* of the feeding roller was 8 cm, the effective length *L* of the feeding roller was 15 cm, the density of ginkgo nut measured in the preliminary test was 0.67 g/cm^3^, the material filling coefficient in the tank *α*_*0*_ was 0.08, the groove radius *r* was 1.25 cm, the cross-sectional area *f*_*q*_ of a single groove was 2.29 cm^2^, the number of grooves was 4, the groove pitch *t* was 6.28, and the driving layer coefficient *λ* was 0.1. Substituting the abovementioned data into the equation, the feed amount per revolution of the feed roller was approximately 33 g. Because the productivity of the sheller was 100–150 kg/h, the speed of the feeding roller could be adjusted from 50–76 r/min, that is, the feeding rate could be adjusted from 28–42 g/s.

### Design of the shelling device

**Fig 4:**
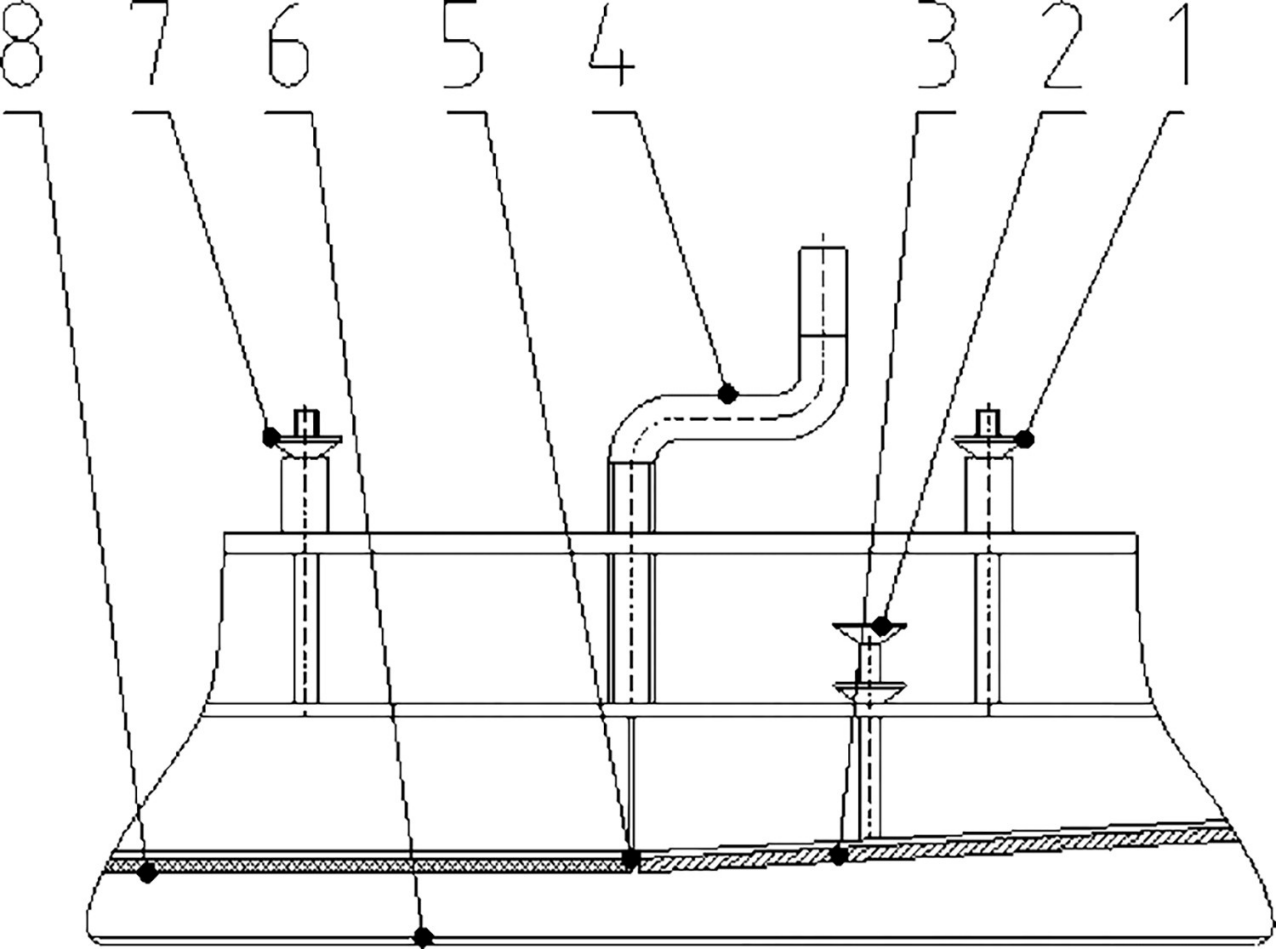
Schematic of the shelling device. 1. Positioning shaft Ⅰ. 2. Adjusting handle Ⅰ. 3. Hard rolling plate. 4. Adjusting handle Ⅱ. 5. Hinge. 6. Chainplate. 7. Positioning shaft Ⅱ. 8. Flexible rolling plate.

The shelling device is the core operation part of the ginkgo nut sheller. Its function is to complete the breaking and shelling processes of the ginkgo nuts. It is the key to obtaining good quality of the shelling operation and separation of shell and kernel while minimizing damage to the ginkgo kernel. The shelling device was composed of hard rolling plate, flexible rolling plate, chainplate, adjusting handles, positioning shafts, and hinge. The schematic is shown in Fig 4. The hard and flexible rolling plates were connected by hinge. The hard rolling plate is an inclined structure. The inclination angle and gap between the hard rolling plate and the chainplate could be adjusted by adjusting the handle. The hard rolling plate was made of food-grade 304 stainless steel. Its length, width, and thickness were 180 mm, 150 mm, and 5 mm, respectively. The flexible rolling plate was parallel to the chainplate. It was made of food-grade PVC material with a Shore hardness of 60 A, which could ensure effective shelling and reduce damage to the kernel. The length, width, and thickness of flexible rolling plate were 150 mm, 150 mm, and 5 mm, respectively. The material of the chainplate was the same as that of the hard rolling plate. It was driven and controlled by an independent motor and a speed regulating mechanism, and the speed of the chainplate could be adjusted in real-time according to the needs of the operation.

### Inclination of the hard rolling plate and chainplate

**Fig 5:**
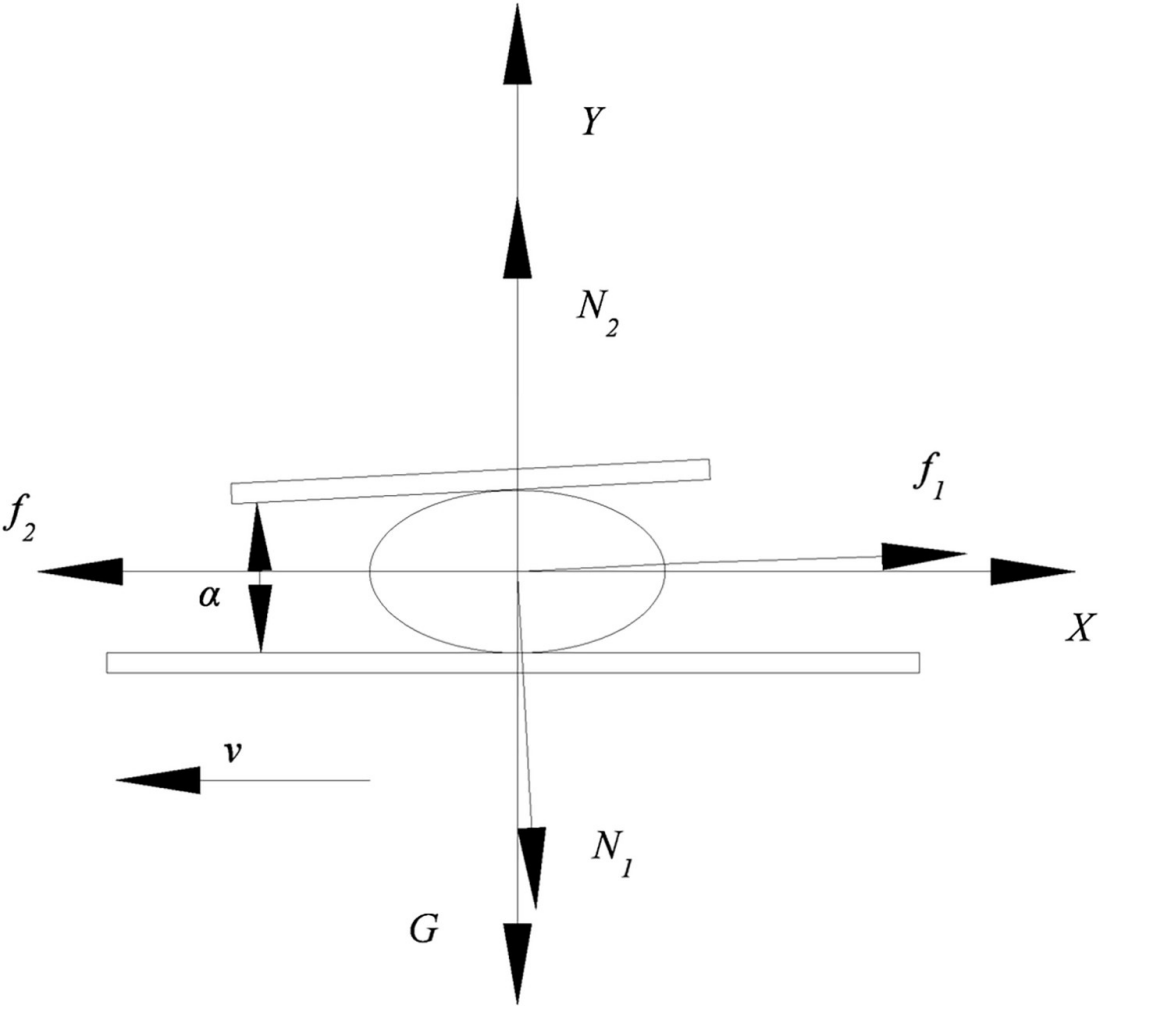
Stress analysis of ginkgo nuts during shell breaking.

The hard rolling plate was inclined and operated with the horizontal chainplate to fully squeeze the ginkgo nuts to be shelled, and the force was gradually increased to obtain a reliable shell breaking effect. The stress produced when the ginkgo nuts passed through the shell-breaking surface formed by the hard rolling plate and chainplate is shown in Fig 5.

Formula (2) must be satisfied in the X direction if ginkgo nuts can pass through the shell-breaking surface smoothly.

$$f_{2}>f_{1}\cos\alpha +N_{1}\sin\alpha$$ (2)

where *f*_*1*_ is the friction force between the ginkgo nut and the hard rolling plate, N;

*f*_*2*_ is the friction force between the ginkgo nut and the chainplate, N;

*N*_*1*_ is the positive pressure of the hard rolling plate on the ginkgo nut, N;

*α* is the included angle between the hard rolling plate and the chainplate, (°).

*f*_*1*_ and *f*_*2*_ can be calculated using Formulas (3) and (4), respectively.

$$f_{1}=\mu N_{1}$$ (3)

$$f_{2}=\mu (N_{2}+f_{1}\sin\alpha )$$ (4)

where *μ* is the friction coefficient between the ginkgo nut, the hard rolling plate, and the chainplate;

*N*_*2*_ is the supporting force of the chainplate to the ginkgo nut, N.

Because the resultant force of the ginkgo nut in the y-axis direction is zero, that is, ∑*F*_*Y*_ = 0, it can be expressed as

$$N_{2}+f_{1}sin\alpha =G+N_{1}cos\alpha$$ (5)

where *G* is the gravity of the ginkgo nut, N, which can be calculated using Formula (6).

G=mg (6)

where *m* is the mass of the ginkgo nut, g;

*g* is the gravitational acceleration, 9.8 m/s^2^.

Using Formulas (2–6), the relationship between the included angle *α* and the other factors can be obtained. It is described in Formula (7).


$$\alpha <\arcsin\frac{\mu mg}{N_{1}}$$ (7)

If *α* > 0, the ginkgo nut is fully squeezed upon passing through the shell-breaking surface, and the force increases gradually, thereby obtaining a reliable shell-breaking effect. Therefore, Formula (8) should be satisfied to ensure shell-breaking effect.


$$0<\alpha <\arcsin\frac{\mu mg}{N_{1}}$$ (8)

#### Chainplate speed

**Fig 6:**
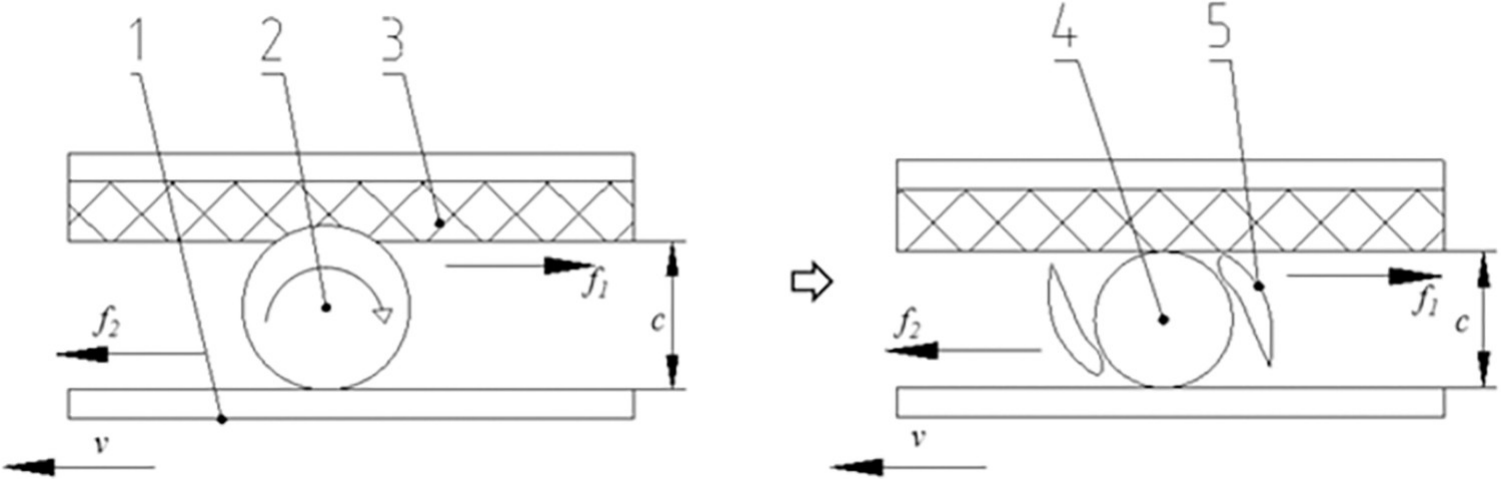
Schematic of the ginkgo nut shelling process. 1. Chainplate. 2. Ginkgo nut. 3. Flexible rolling plate. 4. Ginkgo kernel. 5. Ginkgo shell.

After the shell-breaking process, the shell racks. Because the shells are tough, it is not easy to break them completely; thus, the shell and the kernel cannot be separated. Therefore, a shelling device was added in the design to improve the shelling rate. The shelling process is shown in Fig 6.

The nuts roll around the longitudinal axis because the chainplate moves forward and the flexible rolling plate is stationary when the cracked ginkgo nuts pass through the shelling surface composed of the flexible rolling plate and the chainplate. The cracked shell is squeezed and rubbed, and the cracks continue to spread [24] until the shell is completely broken. Finally, the shell is separated from the kernel. It was found that the speed of the chainplate has an important impact on the shelling quality. In the test, a good operation was obtained when the chainplate speed was 0.3–0.5 m/s.

#### Shelling gap

**Fig 7:**
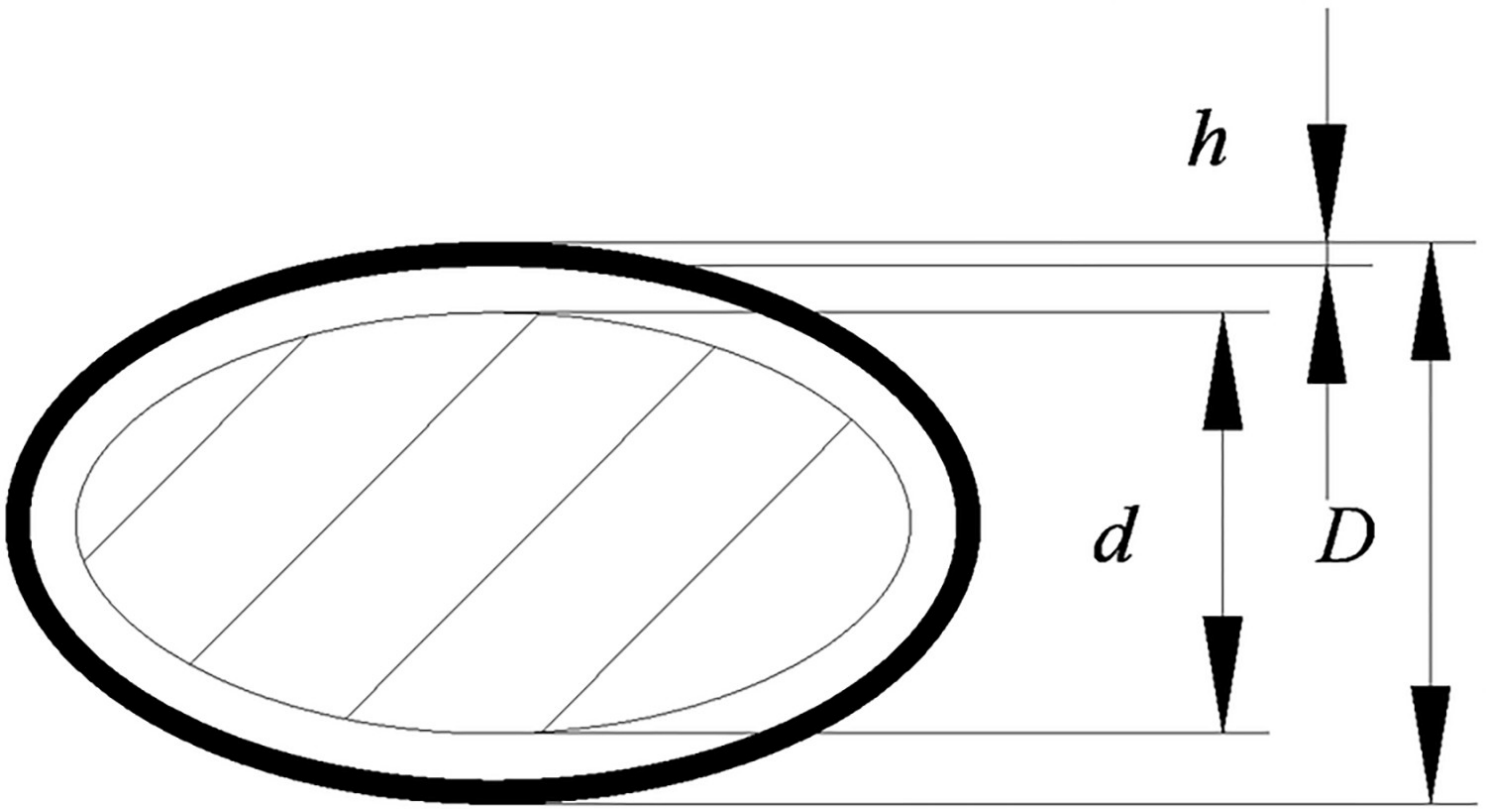
Longitudinal section of the ginkgo nut.

It could be seen in the equipment operation process that the gap between the flexible rolling plate and the chainplate (shelling gap) has an important impact on the quality of the shelling operation. Because the ginkgo nuts are placed on the chainplate in the thickness direction after entering the shelling operation surface, the damage rate of the kernel increases when the shelling gap is small. The shelling rate reduces when the shelling gap is large. The longitudinal section of the ginkgo nut is shown in Fig 7. To ensure that the shell is cracked and the kernel is not broken, the relationship between the shelling gap and ginkgo size should follow Formula (9).

d+2h≤c<D (9)

where *c* is the shelling gap, mm;

*d* is the kernel thickness, mm;

*D* is the nut thickness, mm;

*h* is the shell thickness, mm.

The measured thickness of the ginkgo nut and kernel were 12.8 mm and 10.5 mm, respectively. The average shell thickness was 0.48 mm, thus, 11.5 mm ≤ *c*<12.8 mm.

### Design of centrifugal impurity removal fan

**Fig 8:**
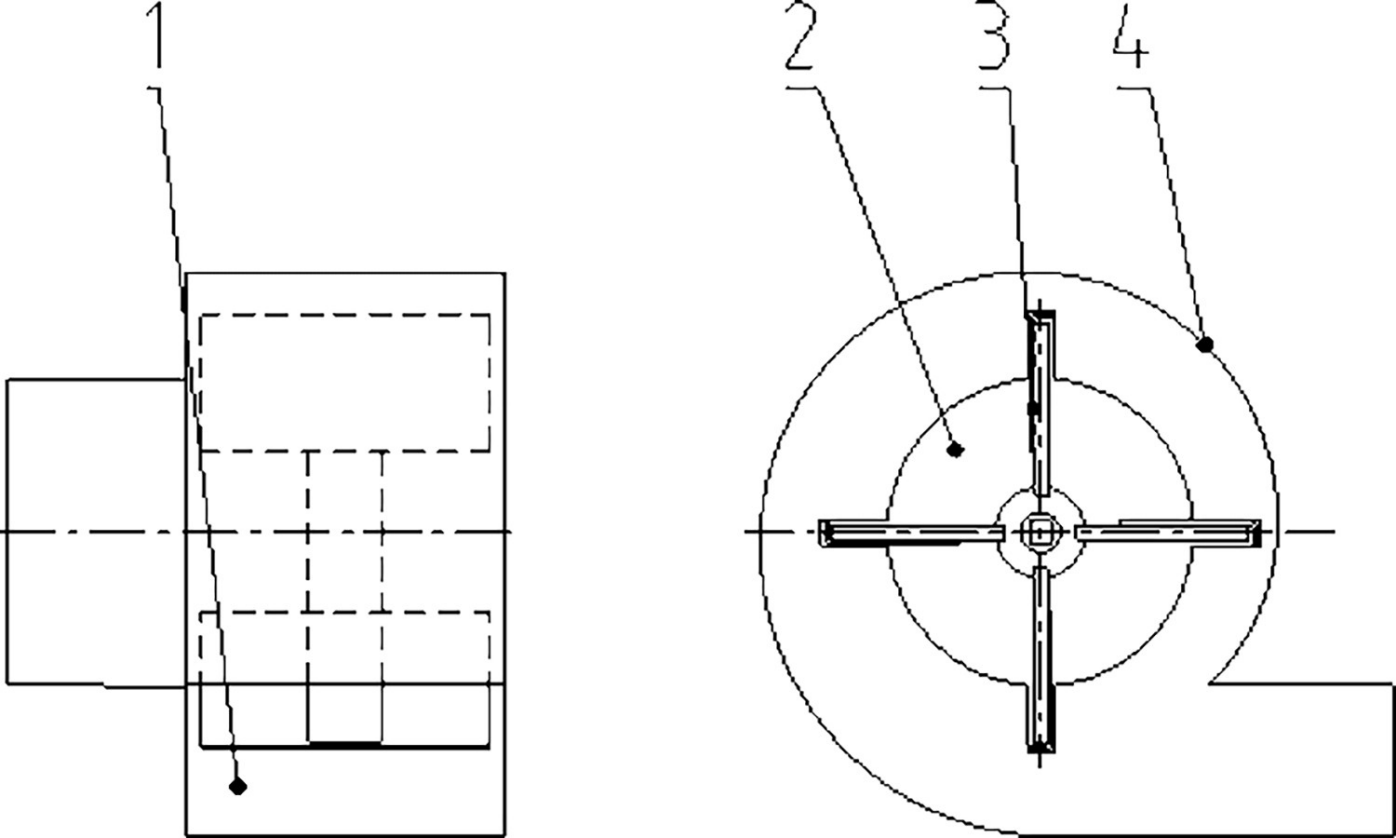
Schematic of the centrifugal fan. 1. Air outlet. 2. Air suction port. 3. Fan impeller. 4. Fan volute.

The suspension speeds of ginkgo shell, nut, and kernel were 6.84–7.20 m/s, 13.90–14.18 m/s, and 15.50–15.76 m/s, respectively [5]. The difference in the suspension speeds can be used in the design of an impurity removal fan [25]. The air velocity at the fan air inlet should be greater than the suspension velocity of the ginkgo shell and less than that of the ginkgo nut. Therefore, in this design, the wind speed range at the fan suction port was set to 7.2–13.9 m/s. The schematic of the centrifugal impurity removal fan is shown in Fig 8.

The outer diameter of the impeller of the centrifugal fan is generally 250–400 mm [26], and in this study, the diameter was 400 mm. The air velocity at the fan outlet [27] can be obtained using Formula (10).

$$v=\alpha v_{c}$$ (10)

where *v* is the wind speed at the air outlet of the fan, m/s;

*α* is the speed increase coefficient, ranging from 1.9 to 3.9; 1.9 was chosen [25];

*v*_*c*_ is the wind speed at the suction outlet, m/s.

Substituting the data, the theoretical value of the wind speed at the outlet was in the range of 13.68–26.41 m/s.

The total pressure of the fan [19] can be obtained using Formula (11).

$$P_{q}=P_{i}+P_{b}$$ (11)

where *P*_*q*_ is the total pressure of the fan, Pa, which can be calculated using Formula (12);

*P*_*i*_ is the static pressure of the fan, Pa, which can be calculated using Formula (13);

*P*_*b*_ is the dynamic pressure of the fan, Pa.

$$P_{i}=\frac{\xi l\rho v^{2}}{2r_{0}g}+\frac{\psi \rho v^{2}}{2g}+\frac{\lambda \rho v^{2}}{2g}$$ (12)

$$P_{b}=\frac{\rho v^{2}}{2g}$$ (13)

where *ξ* is the airflow friction factor; the value used was 0.35 [25];

*l* is the length of the suction pipe; the design value was 0.2 m;

*ρ* is the air density; the value used was 1.293 kg/m^3^;

*r*_*0*_ is the hydraulic radius; the value used was 0.038 m [25];

*g* is the gravity acceleration; the value used was 9.8 m/s^2^;

*ψ* is the resistance coefficient of the pipeline to the airflow; the value used was 0.35 [25];

*λ* is the resistance coefficient of the air inlet and outlet of the fan; the value used was 0.6 [25].

Substituting the data, the calculated total pressure *P*_*q*_ of the fan was 46.04–171.58 Pa. The relationship between the fan speed *n* and fan total pressure *P*_*q*_ is described in Formula (14).

$$n=\frac{60}{\pi D}\sqrt{\frac{P_{q}g}{\varepsilon \rho }}$$ (14)

where *D* is the outer diameter of the impeller of the centrifugal fan, mm;

*ε* is the calculation coefficient; the value range is 0.35–0.4; the value used was 0.4 [26].

The calculated fan speed was 1604–3078 r/min. Based on a preliminary test, the ginkgo shell was completely removed when the fan speed reached 1650 r/min.

## Experiment

### Materials and equipment

The ginkgo nut shelling experiment was conducted in the East Laboratory of Nanjing Institute of Agricultural Mechanization, Ministry of Agriculture and Rural Affairs. The test object species was “Da Fo Zhi” produced in Taixing, Jiangsu. The kernel was full, soft, and easy to break. Prior to the experiment, the ginkgo nuts were cleaned to remove stems, leaves, and other debris. In addition, they were graded based on the external dimensions to obtain clean and uniform size ginkgo nuts. The instruments and equipment used in the test mainly included a Vernier caliper, electronic balance, electronic platform scale, frequency converter, tachometer, tape measure, and stopwatch.

### Test factors and indicators

During the shelling process of ginkgo nuts, many factors can affect the performance of the sheller. To obtain better working quality, the feeding rate, chainplate speed, and shelling gap were selected as the test factors, based on a preliminary design and single-factor test. Taking the shelling rate and damage rate as the main evaluation indicators, a study on the shelling of ginkgo nut was conducted. The feeding rate and chainplate speed were controlled using an independent frequency converter, and the shelling gap was adjusted using an adjustment handle. Because there is no relevant technical standard for shelling of ginkgo nut, this research refers to the People’s Republic of China machinery industry standard "Test Method for Peanut Sheller" (JBT 5688.2–2007) for the test and checking of samples. During the test, the ginkgo nuts were continuously and evenly fed using the feeding device, and sampling began after 10 min of operation and achieving a stable operation. In each sampling, the sample taken for measurement was not less than 1000 g. The total weights of the kernel, unshelled kernel, and damaged kernel in the sample were determined. Each set of the shelling test was repeated three times, and the average value was determined [28, 29]. Each operation index was calculated using the following equations:

$$S=\frac{N_{0}}{N_{0}+N_{w}}$$ (15)

where *S* is the shelling rate, %;

*N*_*0*_ is the total weight of kernel in the sample, g;

*N*_*W*_ is the total weight of unshelled kernel in the sample, g.

$$D=\frac{N_{p}}{N_{0}}$$ (16)

where *D* is the damaged rate, %;

*N*_*p*_ is the total weight of the damaged kernel, g.

### Experimental design and methods

The response surface test was conducted by considering the shelling rate S and damaged rate D as the response values, and the feeding rate A, chainplate speed B, and shelling gap C as the influencing factors. Using the three-factor quadratic regression test design scheme [30, 31], an optimal combination of three main parameters affecting the shelling and damaged rates was obtained. The test factors and coding levels are shown in Table 1.

**Table 1:** Factors and levels of response surface test.

Levels	Factors		
	Feeding rate *A*, (g/s)	Chainplate speed *B*, (m/s)	Shelling gap *C*, mm
-1	30	0.3	11.5
0	35	0.4	12.0
1	40	0.5	12.5

Design-Expert software was used to conduct the experiment based on the Box-Behnken central combinatorial design theory. There was a total of 17 experimental points, including 5 zero-point estimation errors and 12 analysis factors. The experimental design and results are shown in Table 2.

**Table 2:** Experiment design and response values.

No.	Factor levels			Response values	
	Feeding rate *A*, (g/s)	Chainplate speed *B*, (m/s)	Shelling gap *C*, mm	Shelling rate *S*, %	Damage rate *D*, %
1	-1	1	0	99.14	5.13
2	1	-1	0	89.17	2.25
3	0	0	0	97.49	5.14
4	0	-1	-1	94.24	7.97
5	0	1	-1	96.76	9.14
6	1	0	-1	93.24	7.04
7	-1	0	1	97.21	3.03
8	0	0	0	96.73	5.27
9	0	1	1	97.95	4.62
10	-1	0	-1	97.83	7.55
11	0	0	0	96.13	4.98
12	1	1	0	96.54	4.66
13	-1	-1	0	96.08	3.74
14	1	0	1	89.16	2.36
15	0	0	0	96.75	5.63
16	0	0	0	97.86	5.49
17	0	-1	1	90.82	1.56

### Establishment of regression model and significance test

Bases on the test results presented in Table 2, multiple regression fittings were performed using Design-Expert 8.0.6 analysis software. The quadratic polynomial regression model of the shelling rate *S* and damaged rate *D* on the three independent variables (*A*, *B*, *C*) was obtained, as shown in Formulas (17–18). The established regression equation was analyzed using variance analysis, and the results are shown in Table 3.


$$S=96.99-2.77A+2.51B-0.87C+1.08AB-0.86AC+1.15BC-1.17A^{2}-0.59B^{2}-1.46C^{2}$$ (17)

$$D=5.30-0.39A+1.00B-2.52C+0.25AB-0.040AC+0.47BC-1.09A^{2}-0.26B^{2}+0.79C^{2}$$ (18)

**Table 3:** Variance analysis of the regression equation.

Source	Shelling rate *S*, %				Damage rate *D*, %			
	Sum of Squares	df	*F*	*P*	Sum of Squares	df	*F*	*P*
Model	148.53	9	29.36	< 0.0001**	68.70	9	71.52	< 0.0001**
*A*	61.33	1	109.10	< 0.0001**	1.23	1	11.55	0.0115*
*B*	50.40	1	89.66	< 0.0001**	8.06	1	75.51	< 0.0001**
*C*	6.00	1	10.68	0.0137*	50.65	1	474.55	< 0.0001**
*AB*	4.64	1	8.26	0.0238*	0.26	1	2.44	0.1625
*AC*	2.99	1	5.32	0.0544	6.400E-003	1	0.060	0.8136
*BC*	5.31	1	9.45	0.0180*	0.89	1	8.37	0.0232*
*A* ^2^	5.77	1	10.27	0.0150*	5.02	1	47.06	0.0002**
*B* ^2^	1.46	1	2.59	0.1513	0.30	1	2.77	0.1403
*C* ^2^	8.99	1	15.99	0.0052**	2.60	1	24.32	0.0017**
Residual	3.93	7			0.75	7		
Lack of Fit	2.06	3	1.47	0.3494	0.47	3	2.30	0.2187
Pure Erro	1.87	4			0.27	4		
Cor Total	152.47	16			69.45	16		
R-Squared	0.9742				0.9892			
Adj R-Squared	0.9410				0.9754			

Note: *P* < 0.01 (highly significant, **); 0.01 ≤ *P* < 0.05 (significant, *).

Table 3 shows that the two regression models were highly significant (*P* < 0.01), and the fitting degrees of the regression equations were high (*P* > 0.05). The determination coefficients *R*^*2*^ of the models were 0.9742 and 0.9892, indicating that the correlations between the predicted and actual values of the models were high and the test errors were small. Therefore, the models can be used to analyze and predict the shelling and damage rates of the ginkgo nut sheller.

Table 3 shows that in the *S* model for the shelling rate, the regression terms *A*, *B*, and *C*^*2*^ had high significant impacts on the model (*P* < 0.01), the regression terms *C*, *AB*, *BC*, and *A*^*2*^ had significant impacts on the model (*P* < 0.05), whereas *AC* and *B*^*2*^ had no significant impact on the model (*P* > 0.05). In the *D* model for the damage rate, the four regression terms *B*, *C*, *A*^*2*^, and *C*^*2*^ had high significant impacts on the model (*P* < 0.01), the two regression terms *A* and *BC* had significant impacts on the model (*P* < 0.05), whereas *AB*, *AC*, and *B*^*2*^ had no significant impact on the model (*P* > 0.05). To make the abovementioned models highly significant and the lack-of-fit items not significant, the significant items were retained and the insignificant items were removed.


$$S=96.74-2.77A+2.51B-0.87C+1.08AB+1.15BC-1.20A^{2}-1.49C^{2}$$ (19)

$$D=5.19-0.39A+1.00B-2.52C+0.47BC-1.11A^{2}+0.77C^{2}$$ (20)

### Analysis of the influences of interactive factors on performance

#### Analysis of the influence of shelling rate

**Fig 9:**
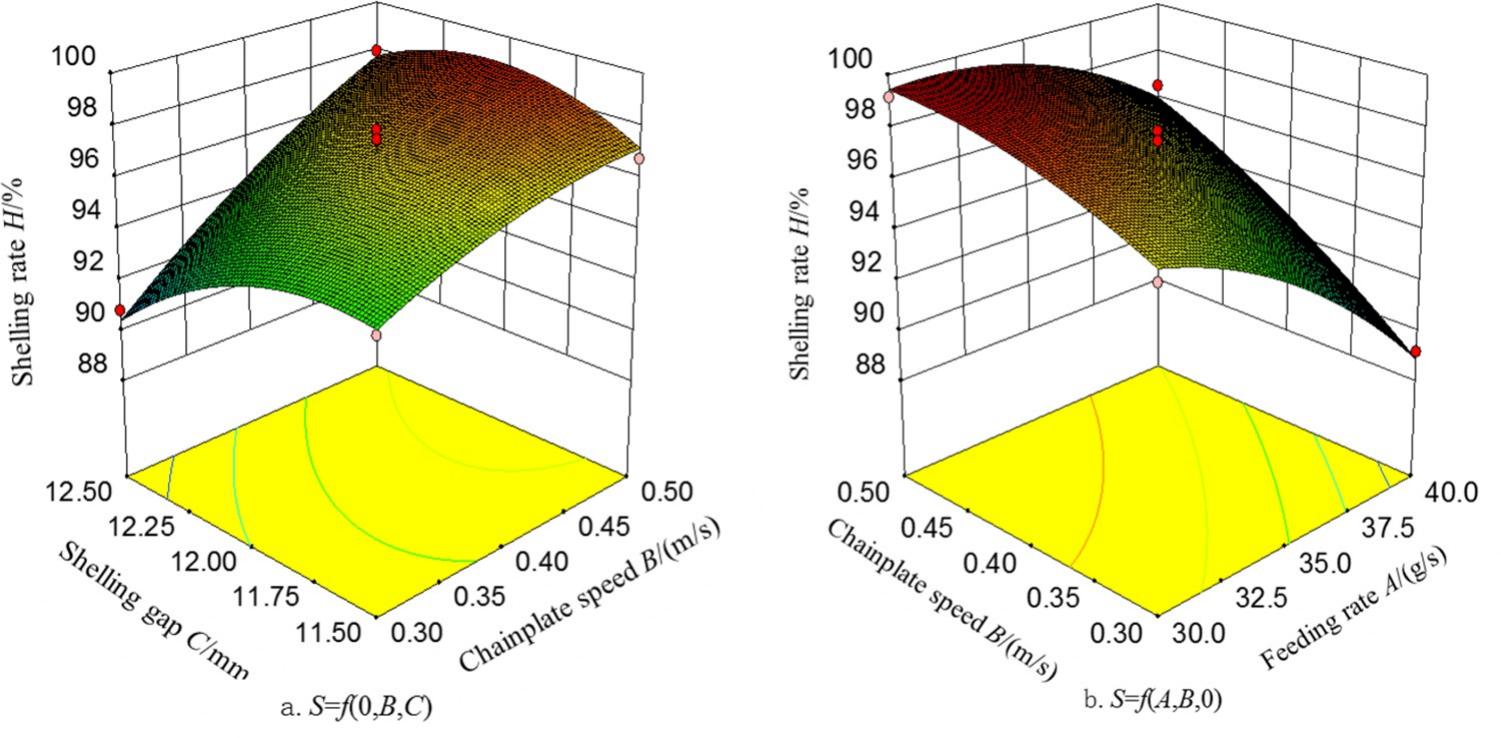
Response surfaces of the factors’ influence of shelling rate.

Based on the coefficient analysis of each regression term in the regression model [32–35], the order of the factors based on their influence on the shelling rate of the ginkgo nut sheller is as follows: feeding rate, chainplate speed, and shelling gap. The order of the factors based on their influence on the damage rate of the ginkgo nut sheller is as follows: shelling gap, chainplate speed, and feeding rate. Design-Expert 8.0.6 analysis software was used to draw response surface and contour maps to analyze the influence of significant interaction of various factors on the shelling rate. Fig 9A. shows the response surface and contour diagram of the interaction between the chainplate speed and shelling gap on the shelling rate when the feeding rate was at the middle level. When the feeding rate was 35 g/s, the shelling rate *S* gradually increased as the speed of the chainplate increased because as the speed of the chainplate increased, the force that squeezed and rubbed the ginkgo nuts increased. The shell tended to crack and break, which was good for the separation of shell and kernel. When the shelling gap was the highest level, the increasing trend of the shelling rate with the increase in the chainplate speed was more apparent. When the shelling gap was the lowest level, the trend was relatively flat. As the shelling gap decreased, the shelling rate increased and then decrease. This was because when the shelling gap decreased, the shells of the ginkgo nuts were strengthened by the action of the hard and flexible rolling plates, which made the shell and kernel easier to separate. Then the shelling rate increased. As the gap continued to decrease, the shelling rate reached its maximum value. When the gap was further reduced, the ginkgo nuts were severely squeezed and broken. Therefore, the shell and the kernel were stuck together and could not be separated, resulting in a reduction in the shelling rate. Fig 9B shows the response surface and contour diagram of the interaction of the feeding rate and chainplate speed on the shelling rate when the shelling gap was at the middle level. When the shelling gap was 12 mm, the shelling rate *S* gradually increased as the feeding rate decreased because as the feeding rate decreased, the number of ginkgo nuts to be shelled per unit time decreased, which caused the extrusion and kneading functions more efficient, and thus improved the shelling rate. When the chainplate speed was the lowest level, the gradual increase of the shelling rate with the decrease in the feeding rate was more apparent. When the chainplate speed was the highest level, the trend was relatively flat.

#### Analysis of the influence of damage rate

**Fig 10:**
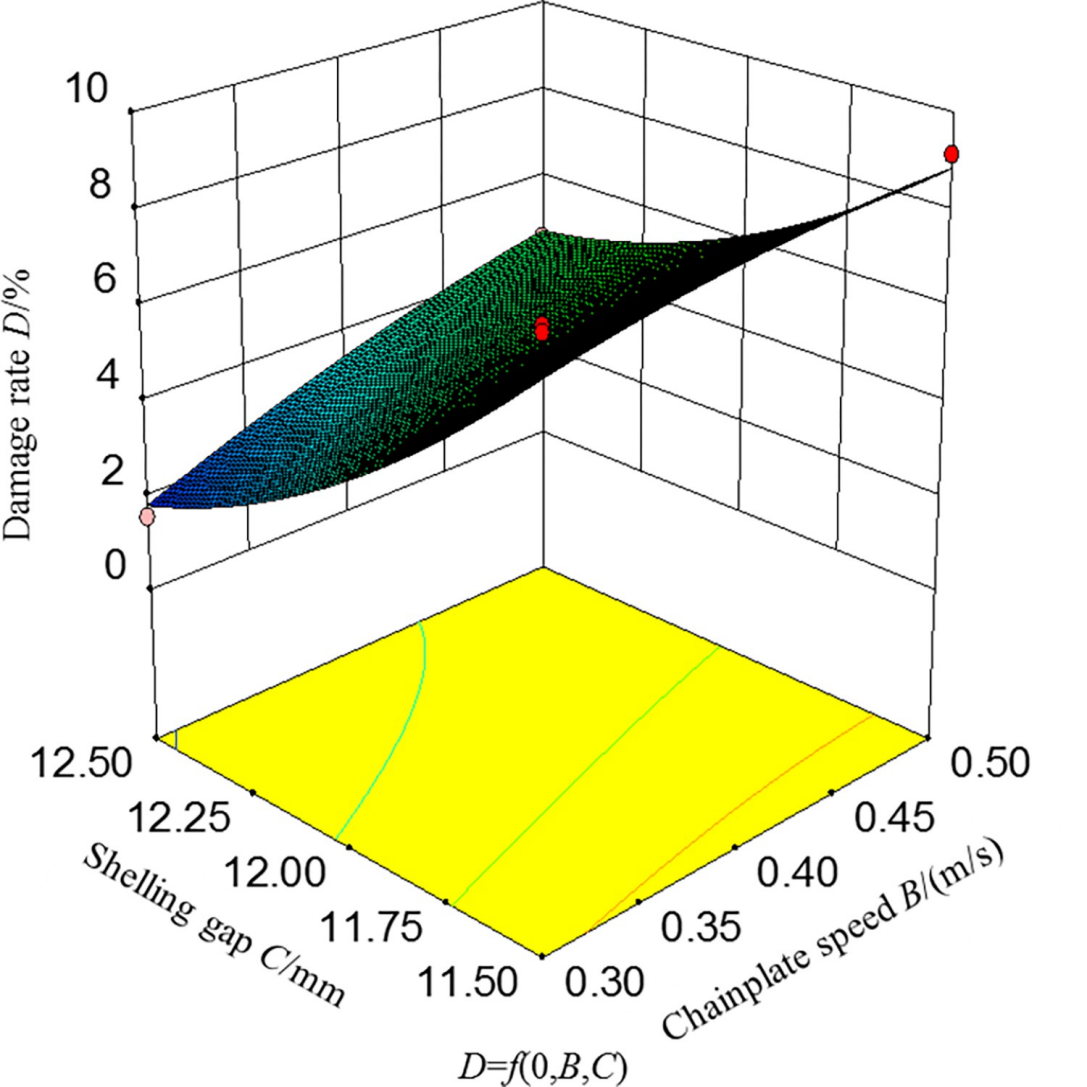
Response surfaces of the factors’ influence of damage rate.

Fig 10. shows the response surface and contour diagram of the interaction between the chainplate speed and shelling gap on damage rate when the feeding speed was at the middle level. When the feeding rate was 35 g/s, the damage rate *D* gradually decreased with the decrease in the chainplate speed and increase in the shelling gap. This is because when the chainplate speed decreased and the shelling gap increased, the force on the ginkgo nuts during the shelling process decreased, thereby reducing the damage rate. When the shelling gap was the highest, the damage rate gradually decreased as the chainplate speed decreased. When the shelling gap was the lowest level, the trend was flat. When the chainplate speed was the lowest level, the gradual decrease in the damage rate as the shelling gap increased was more apparent. When the chainplate speed was the highest level, the trend was relatively flat. In addition, the impact of the shelling gap on the damage rate was greater than that of the chainplate speed. It indicates that when the feeding speed was at the middle level, the impact of the shelling gap on the damage rate was more significant than that of the chainplate speed.

### Parameter optimization and verification

Because of the different influences and interactions of the various factors on the shelling and damage rates, it was necessary to comprehensively consider and perform multi-objective optimization to optimize the operational performance of the ginkgo nut sheller. The double objective function mathematical model for the shelling rate *S* and damage rate *D* was established with the highest shelling rate and the lowest damage rate as the optimization objective. The constraint conditions for each parameter variable were set as shown in Formula (21). The optimal solutions when the shelling rate *S* was the highest and the damage rate *D* was the lowest were as follows: the feeding rate was 30.22 g/s, the chainplate speed was 0.48 m/s, and the shelling gap was 12.32 mm. This resulted in an S value of 99.27% and D value of 4.24%.


$$\left\{ \begin{array}{l} \max H(A,B,C)\\ \min D(A,B,C)\\ -1\leq A\leq 1\\ -1\leq B\leq 1\\ -1\leq C\leq 1 \end{array}\right.$$ (21)

Because the response interview did not include the abovementioned optimal solution combination, the parameters were adjusted based on the actual operation to verify the accuracy of the model prediction and the reliability of the optimization results. The optimal solution combination was corrected; thus, the feeding rate was 30.2 g/s, the chainplate speed was 0.48 m/s, and the shelling gap was 12.3 mm. Then, an experimental verification using a total of five tests was conducted, and the average of the results was calculated [36–38]. The test results are presented in Table 4.

**Table 4:** Comparison between model predictions and measured values.

Parameter	Shelling rate *S*, %	Damage rate *D*, %
Test value	98.02	4.45
Predicted value	99.27	4.24
Relative error	1.26	4.95

Table 4 shows that the relative errors between the test results of the ginkgo nut shelling operation and the model results were small (both less than 5%). The test results were close to the predicted values, which indicates that the optimized regression models of the abovementioned parameters had high reliability. Using this parameter combination, that is, a feeding rate of 30.2 g/s, a chainplate speed of 0.48 m/s, and a shelling gap of 12.3 mm, the resulting shelling rate was 98.02% and the damage rate was 4.45%.

## Discussion

### Multifunctional sheller resolved the shelling problem of ginkgo nut

The existing ginkgo nut sheller only has the shell-breaking parts, but has no shelling parts. Therefore, the shell and kernel cannot be separated completely, and the operation quality needs to be improved. In response to this situation, the structure of core operating components was innovated and a multifunctional ginkgo nut sheller different from the existing models was designed. The sheller can perform multiple processes, such as shell breaking in the hard rolling plate, shelling in the flexible rolling plate, and separation of the shell from the kernel in one operation. It can realize high-efficiency and low-loss shelling of ginkgo nut. In addition, an experiment was conducted to determine the influence of the main factors of the sheller on shelling and damage rates. Then, a combination of the operational parameters was optimized to improve the operation quality.

### RSM simplified the investigation

RSM and other existing methods (orthogonal design, factorial design, etc.) can be used for experimental optimization design. It can obtain good and valid information with less experimental cost and time. The effects of factor interactions can be explored in depth and optimal conditions can be obtained. At the same time, RSM takes the response value of the system as a function of one or more factors, and uses graphic technology to express the functional relationship, which is more intuitive and more efficient. Compared with RSM, the optimal value obtained by orthogonal design method is only a combination of the levels used in the test, and the optimal result will not exceed the range of the levels taken, and the accuracy of the results is not high. Factorial design requires a lot of experiments, so it is time-consuming and inefficient. In the study of ginkgo shelling, it is necessary to analyze the influence of multiple test factors and their interaction on the operation quality. RSM can optimize the related operation parameters accurately and efficiently, which satisfies the needs of this study well. However, it should be noted that the number of parameters we studied is limited, and more factors can be added in the future to conduct a more in-depth study on ginkgo nut shelling.

### Provide a practical sheller for ginkgo producers

In this study, a multifunctional ginkgo nut sheller was designed. Compared with the existing equipment, the sheller has innovative structure and better operation performance, which can meet the actual operation requirements, and provide a practical sheller for ginkgo producers. Policy-makers can increase support for the research results, formulate appropriate policies, speed up the application process, so that more ginkgo producers can benefit. At the same time, the application of this research results is beneficial to promote the development of ginkgo industry.

## Conclusion

The multifunctional ginkgo nut sheller has good operation performance, which can provide a practical sheller for ginkgo producers to mechanize shelling. The experimental results showed that the order of importance of the factors on the shelling rate was as follows: feeding rate, chainplate speed, and shelling gap. The order of importance of the factors on the shelling rate was as follows: shelling gap, chainplate speed, and feeding rate. The results of the interaction analysis showed that the interaction between the chainplate speed and shelling gap had the highest significant effect on the shelling rate, followed by the interaction between the feeding rate and chainplate speed. The interaction between the chainplate speed and shelling gap had a significant effect on the damage rate. The interaction between other factors had no significant effect on the shelling and damage rates. The optimal combination of the parameters was as follows: the feeding rate was 30.2 g/s, the chainplate speed was 0.48 m/s, and the shelling gap was 12.3 mm. The theoretical values were as follows: the shelling rate was 99.27% and the damage rate was 4.24%. The optimal combination was used to perform multi-batch experiments. The shelling rate was 98.02% and the damage rate was 4.45%. The relative error between the test and theoretical values was less than 5%, indicating that the models were reliable.

## Supporting information

S1 Table Factors and levels of response surface test. The table lists three levels of parameter setting for the three factors in the test.
(XLSX)

S2 Table Experiment design and response values. The table lists all 17 experimental points.
(XLSX)

S3 Table Variance analysis of the regression equation. Note: *P* < 0.01 (highly significant, **); 0.01≤*P* < 0.05 (significant, *).
(XLSX)

S4 Table Comparison between model predictions and measured values. (XLSX)

S1 Fig Schematic of the chainplate ginkgo nut sheller. 1. Positioning shaft Ⅰ. 2. Adjusting handle Ⅰ. 3. Hard rolling plate. 4. Adjusting handle Ⅱ. 5. Hinge. 6. Chainplate. 7. Positioning shaft Ⅱ. 8. Flexible rolling plate. 9. Discharge guide plate Ⅰ. 10. Discharge guide plate Ⅱ. 11. Impurity removal fan. 12. Discharge guide plate Ⅲ. 13. Ground wheel. 14. Motor Ⅰ. 15. Motor Ⅱ. 16. Frame. 17. Feeding guide. 18. Feeding roller. 19. Hopper.
(TIF)

S2 Fig Ginkgo nut triaxial size. (TIF)

S3 Fig Schematic of the feeding roller. 1. Shaft. 2. Outer roller. 3. Groove.
(TIF)

S4 Fig Schematic of the shelling device. 1. Positioning shaft Ⅰ. 2. Adjusting handle Ⅰ. 3. Hard rolling plate. 4. Adjusting handle Ⅱ. 5. Hinge. 6. Chainplate. 7. Positioning shaft Ⅱ. 8. Flexible rolling plate.
(TIF)

S5 Fig Stress analysis of ginkgo nuts during shell breaking. (TIF)

S6 Fig Schematic of the ginkgo nut shelling process. 1. Chainplate. 2. Ginkgo nut. 3. Flexible rolling plate. 4. Ginkgo kernel. 5. Ginkgo shell.
(TIF)

S7 Fig Longitudinal section of the ginkgo nut. (TIF)

S8 Fig Schematic of the centrifugal fan. 1. Air outlet. 2. Air suction port. 3. Fan impeller. 4. Fan volute.
(TIF)

S9 Fig Response surface and contour diagram of the interaction between the chainplate speed and shelling gap on the shelling rate. (TIF)

S10 Fig Response surface and contour diagram of the interaction between the feeding rate and chainplate speed on the shelling rate. (TIF)

S11 Fig Response surfaces of the factors’ influence of damage rate. (TIF)
