# Supplementary figures and images for: Design and optimization of sheller for ginkgo nut: A study about multifunctional ginkgo nut sheller
Source: PLoS One. 2022 Oct 27;17(10):e0276139. doi: 10.1371/journal.pone.0276139 (PMC9612558; doi:10.1371/journal.pone.0276139)

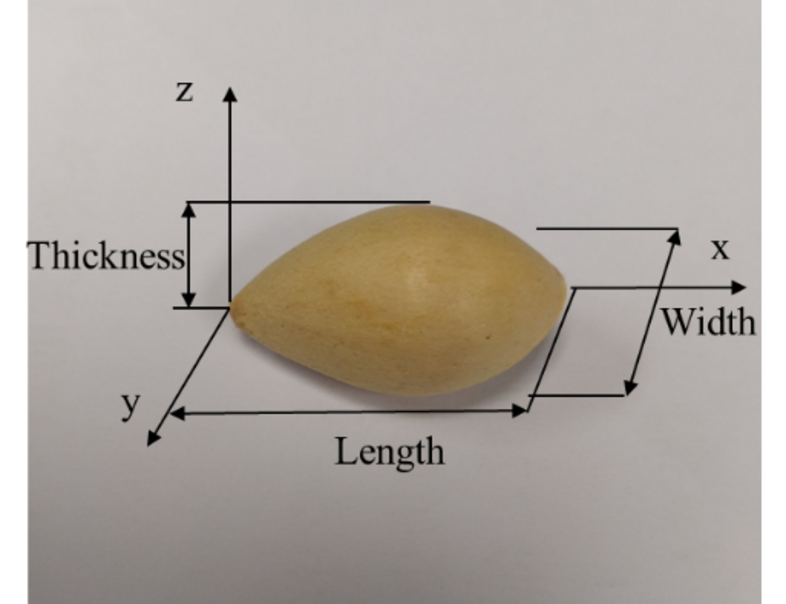

Supplement: S2 Fig — (TIF) [file pone.0276139.s006.tif]

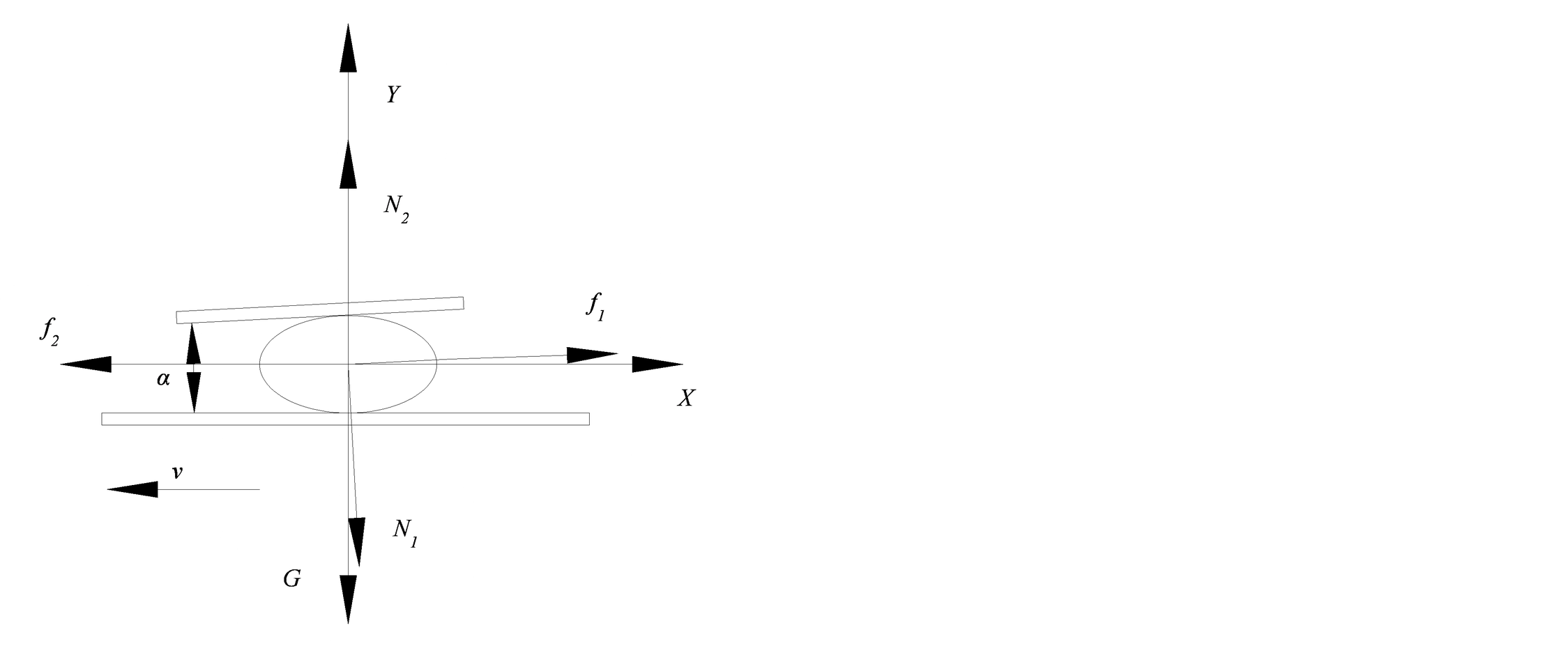

Supplement: S5 Fig — (TIF) [file pone.0276139.s009.tif]

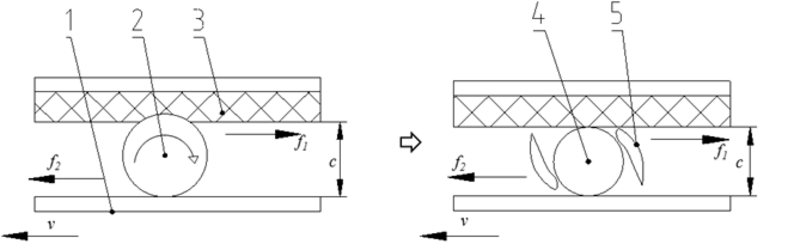

Supplement: S6 Fig — 1. Chainplate. 2. Ginkgo nut. 3. Flexible rolling plate. 4. Ginkgo kernel. 5. Ginkgo shell. (TIF) [file pone.0276139.s010.tif]

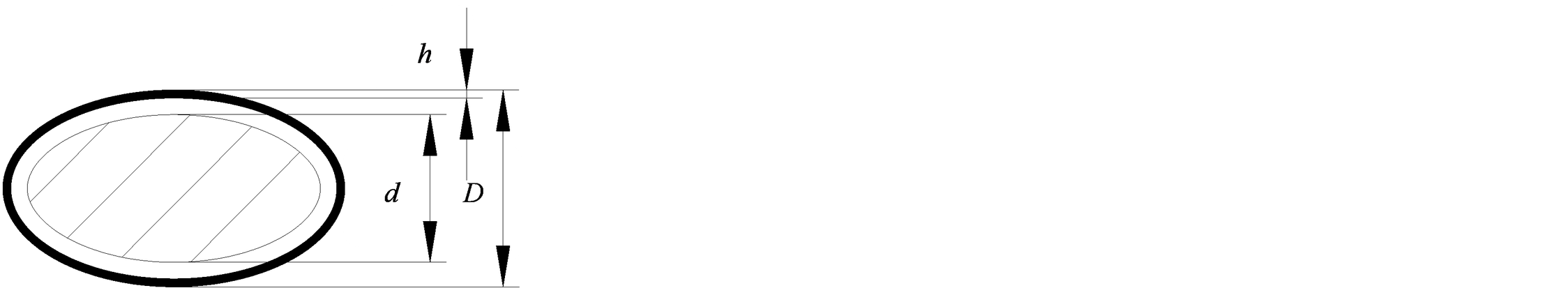

Supplement: S7 Fig — (TIF) [file pone.0276139.s011.tif]

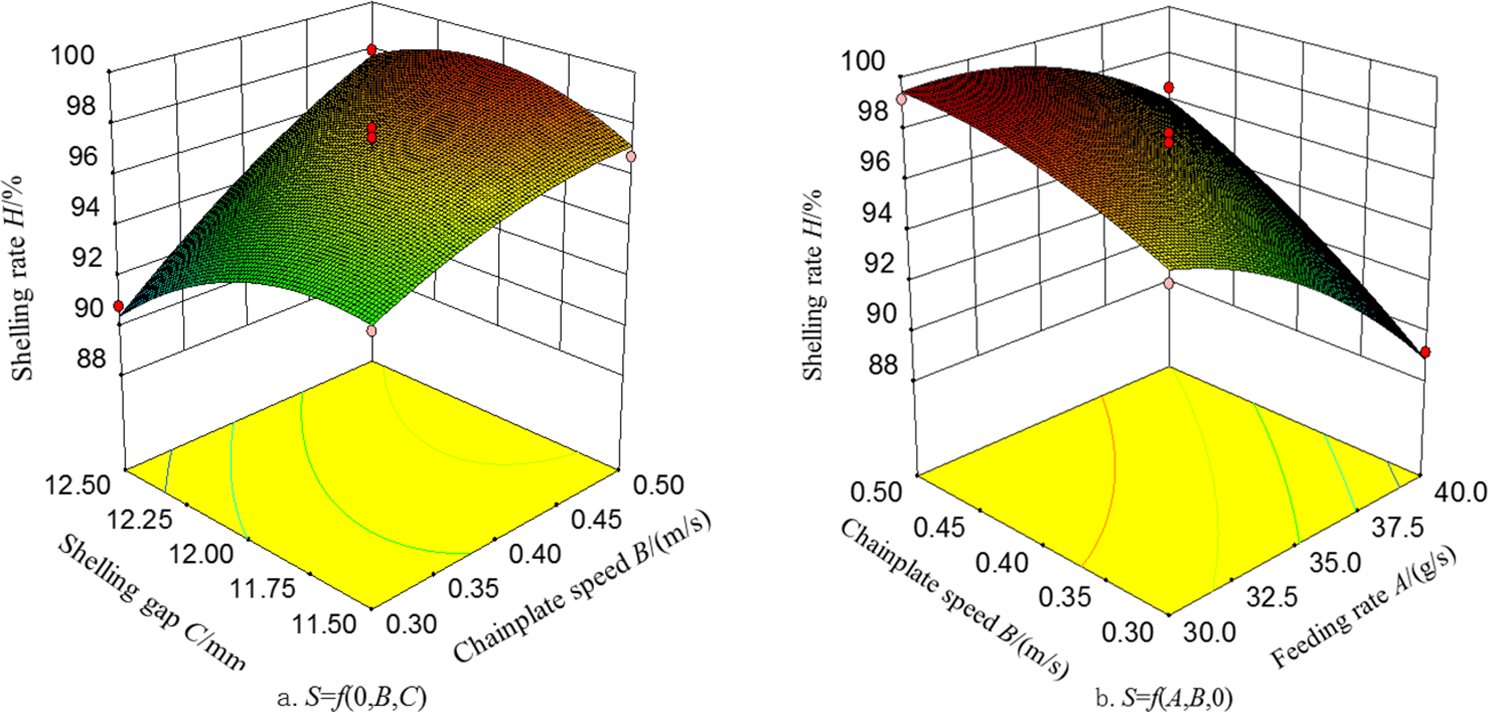

Supplement: S9 Fig — (TIF) [file pone.0276139.s013.tif]

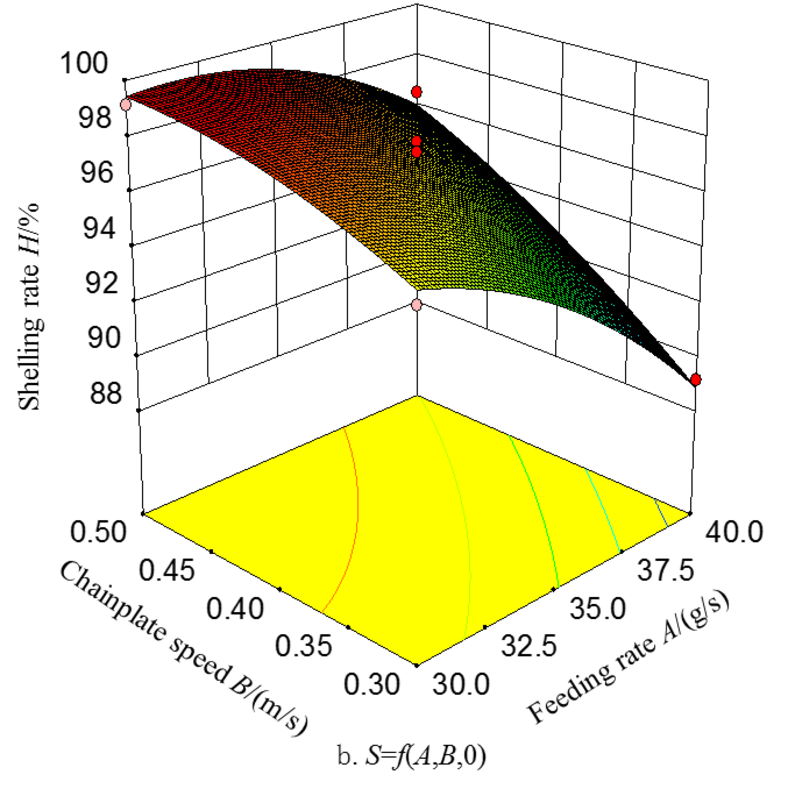

Supplement: S10 Fig — (TIF) [file pone.0276139.s014.tif]

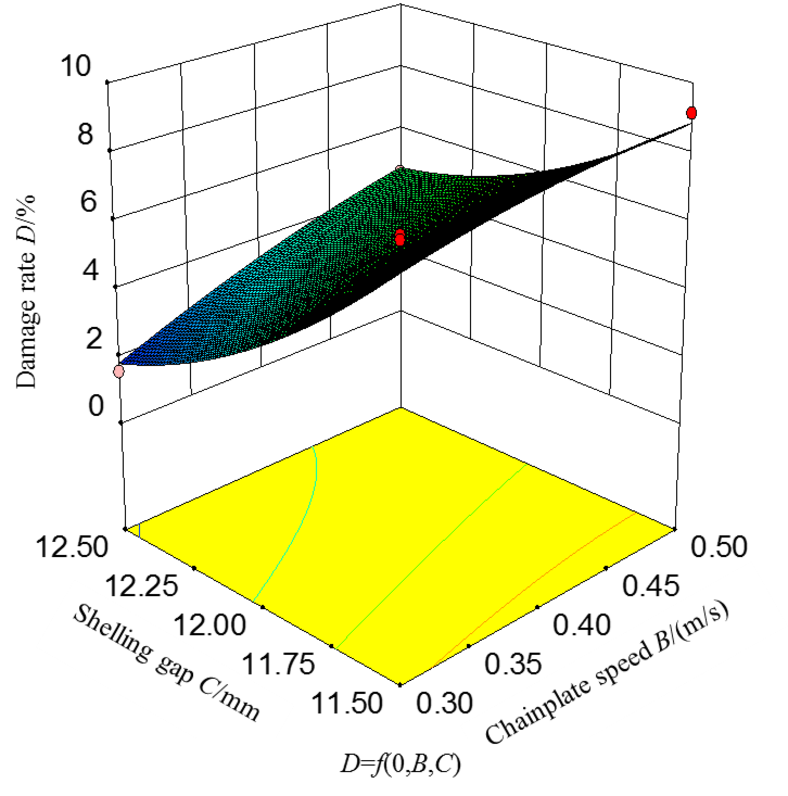

Supplement: S11 Fig — (TIF) [file pone.0276139.s015.tif]
